# Prevalence and determinants of overweight/obesity among under-five children in sub-Saharan Africa: a multilevel analysis

**DOI:** 10.1186/s12887-022-03645-z

**Published:** 2022-10-08

**Authors:** Belete Achamyelew Ayele, Sofonyas Abebaw Tiruneh, Asnakew Achaw Ayele, Melkamu Aderajew Zemene, Ermias Sisay Chanie, Habtamu Shimels Hailemeskel

**Affiliations:** 1 Amhara Regional Health Bureau, Wogeda Primary Hospital, Wogeda, Amhara Ethiopia; 2 Department of Public Health, College of Health Sciences, Debre Tabor University, Debre Tabor, Ethiopia; 3 Department of Clinical Pharmacy, School of Pharmacy, College of Medicine and Health Science, University of Gondar, Gondar, Ethiopia; 4 Department of Pediatrics and Child Health Nursing, College of Health Sciences, Debre Tabor University, Debre Tabor, Ethiopia; 5 Department of Neonatal Nursing, College of Health Sciences, Debre Tabor University, Debre Tabor, Ethiopia

**Keywords:** Under-five children, Overweight, Obesity, Sub-Saharan Africa

## Abstract

**Introduction:**

Childhood obesity has become a major public health problem for both developed and developing nations. It is uncommon to find under-nutrition in many low and middle-income countries; as well, obesity is a double burden in these settings. This study aimed to investigate the pooled prevalence of overweight /obesity among under-five (under-5) children in sub-Saharan Africa (SSA).

**Methods:**

Data were accessed from the recent nationally representative demographic and health survey datasets from 33 SSA Countries. A total of 192,132 under-five children were recruited for this study. The pooled prevalence of overweight /obesity among under-5 was done using random-effects meta-analysis command. Multivariable multi-level mixed-effects logistic regression analysis was used to identify determinants for the prevalence of under-5 overweight and/or obesity. A P-value less than 0.05 was used to declare statistical significance.

**Results:**

The pooled prevalence of overweight /obesity among under-5 was 5.10% (9% CI: 4.45 – 5.76) in SSA. South Africa region (8.80%, 95% CI: 4.18 – 13.42) had a higher prevalence of under-5 overweight and/or obesity followed by the East Africa region. Male under-5 children (adjusted odds ratio (AOR) = 1.09, 95 confidence interval (CI): 1.02 – 1.25), Larger birth weight under-5 children (AOR = 1.39, 95% CI: 1.26 – 1.54), under-5 children aged older two to three years (AOR = 0.85, 95% CI: 0.76 – 0.94), under-5 children born from educated mothers (secondary and above) (AOR = 1.12, 95% CI: 1.01 – 1.25), and under-5 children living in the West Africa (AOR = 0.67, 95% CI: 0.56 – 0.81) and South Africa (AOR = 1.87, 95% CI: 1.09 – 3.21) were significant determinants for under-5 overweight and/or obesity.

**Conclusion:**

Childhood obesity is becoming a great challenge and double burden in developing nations. In SSA Africa 1 in 20 under 5 children were overweight and/or obese. Male under-5 children, older aged, under-5 children born from educated mothers, and under-5 children living in the South Africa region were at higher risk for developing overweight and/or obesity. Thus, SSA countries should implement early to pause these consequences preventing the double burden of undernutrition.

## Introduction

Childhood obesity has become a major public health problem for both developed and developing nations [1]. Under-5 children died due to overweight and obesity than underweight in most of the world's population. Despite this, about 39 million children under the age of 5 years were overweight or obese in 2020 [2]. It is not uncommon to find under-nutrition in many low- and middle-income countries, as well as obesity existing within the same Country, the same community even within the same household in these settings [3]. The prevalence of under-5 (under-5) overweight and obesity in European state member Countries ranges from 1 to 28.6% [4, 5]. Middle-income countries shared more than three-quarters (79%) of under-5 overweight and obesity [8]. In Southern and Northern Africa, under-5 overweight or obesity is 13% and 10.6%, respectively [6].

In the African region, especially in urban settings, the prevalence of under-5 overweight and/or obesity is a threatening condition. Between 2000 to 2015 years, it increased by more than 50% [7]. Sub-Saharan African (SSA) Countries are challenging for under-5 children with undernutrition problem. In parallel with under-nutrition SSA Countries are experiencing an increasing double burden of under-5 overweight and obesity [8, 9]. Paradoxically, as World Health Organization assessed at primary health care facilities [10], under-5 overweight and/or obesity are commonly observed in previously disadvantaged and lower socio-economic groups. Surprisingly, a study done in South Africa showed that among the obese group, 68.4% were stunted before [11]; about one-fourth of obese mothers during pregnancy, accelerating the risk of transmission of overweight and obesity in their offspring [9]. The cause of overweight and obesity is complex, but there are suggested contributors, such as poverty, physical inactivity, poor diet, population-level of education, mal food practice, maternal health and family size are some of the factors for childhood overweight and/or obesity increasing [10, 12, 13].

If a child is overweight / obese is left not managed early in childhood, it will increase the risk of non-communicable disease late in childhood and adolescent life. Childhood obesity can greatly affect children’s developmental and physical health, social, and emotional well‑being, and self-esteem; as well as risk for many co‑morbid conditions like metabolic, cardiovascular, orthopedic, neurological, hepatic, pulmonary, and renal disorders [13–16]. Childhood obesity is also associated with poor academic performance and a lower quality of life experienced by the child [16]. Furthermore, under-5 obesity incurs direct health care and indirect costs that influence the family's economic activities and subsequently countries at large [17, 18],

Under-5 overweight and obesity is a highly preventable non-communicable disease. Despite this, in SSA Countries, the bulk of research is dedicated to undernutrition which is still caused by food insecurity, but silently shifts nutritional status to over nutrition (overweight and/or obesity) [19]. To the best of our knowledge, in SSA the pooled prevalence of under-5 overweight and/or obesity is not yet studied. Unless, SSA countries put together, the strengthened effect to curb this problem, these countries will not achieve the Sustainable Development Goals (SDGs) to curb under-5 overweight and/or obesity by 2030 [20]. Therefore, this study aimed to investigate the pooled prevalence and determinants of overweight /obesity among under-5 children in SSA using nationally representative demographic and health survey datasets. The findings are expected to provide new insight and updated information regarding the problem for health policymakers and stakeholders at large to curb the problem.

## Methods and materials

### Data sources

The data for this study was extracted from the recent standard DHS (2010–2020) datasets of 33 SSA Countries. Standard Demographic and Health Surveys (DHSs) are nationally representative and population-based surveys collected through uniform questionnaires and manuals that are comparable across countries. The data were collected using multi-stage stratified, cluster sampling techniques for each country. The details of the recorded data available at https://dhsprogram.com/

### Populations and samples

The source population for this study was all under-5 children aged less than 60 months in five years preceding the survey year in 33 SSA Countries. The data for this study were extracted from the children's (KR) record file. In the KR file, all children who were born in the last five years preceding the survey in the selected enumeration area (EAs). A total of 192,132 under-5 children were included from 33 SSA countries.

### Study variables

The dependent variable for this study was variable declared based on new World Health Organization (WHO) child growth monitoring charts. Thus, overweight and/or obesity is declared if the child's weight-for-height z-score is above plus 2 (+ 2.0) standards deviations (SD) above the mean [11], otherwise not overweight and/or obese. The independent variables were thematized as individual and region or province-level variables. The individual-level variables were the sex of the child (male, female), the plurality of birth (single, multiple), birth size at birth (low birth weight, average birth weight, and larger than average), child age in years (less than two years, two–three years, and above three years), exclusive breastfeeding (yes, no), preceding birth interval (≥ 24 months, 18 -23 months, and < 18 months), age of the mother (15—19 years, 20—34 years, and ≥ 35 years), mother education (no education, primary education, and secondary and above), father education (no education, primary education, and secondary and above), mother’s occupation (not working, working), father occupation (not working, working), family size (< 4, ≥ 4 +), wealth index (Poor, Middle, and Rich), place of delivery (institutional, home). The region or provinces were SSA Sub-regions (East Africa, West Africa, Central Africa, and South Africa), Country income (Upper middle income, lower middle income, and Low income), and Residence (Urban, Rural).

### Data management and analysis

The data were cleaned, coded, and extracted using Microsoft Excel and STATA version 16/MP software. Sample weighting was performed for each country before further analysis. To declare Under-5 overweight/obesity, weight, height, age, and sex of the child were used from the KR DHS dataset file. Using the parameters weight, height, age, and sex of the child the weight for height (WHZ) z-score was analyzed using the WHO Anthro software. After WHZ score was generated, it was categorized using the cut point above plus 2 (+ 2.0) standards deviations (SD) to declare under-5 overweight/obesity.

### Multilevel mixed-effects logistic regression modeling

The pooled prevalence of under-5 overweight and/or obesity conducted using meta-analysis SATTA command presented as a forest plot. The DHS data were collected in a multistage or hierarchical approach, this hierarchical nature could violate the assumption of data independence in statistics. Therefore, ignoring this data dependency would affect the true effect size of our analysis. Children were nested within the administrative region or province of each Country and regions nested in each Country. Thus, children who live within the same administrative region had the same risk of developing the event of interest as children who live in another administrative region in each Country. This scenario needs accounting for the data dependency using multilevel modeling. Therefore, we used the mixed-effect logistic regression analysis to control administrative region dependency. The assumption of administrative region correlation was checked using the Intra-class Correlation Coefficient (ICC). If the ICC value greater than 0.25 (16) was reasonable to fit the multilevel mixed-effects logistic regression model. In mixed-effect logistic regression modeling, both fixed effect and random effect variable was considered. In this case, the administrative region for each Country was considered a random variable. Since the model was nested in nature Likelihood Ratio (LR) test was used for model compression. Median Odds Ratio (MOR) was employed to observe the heterogeneity between administrative regions. Percentage Change Variation (PCV) measures the total variation attributed by individual and administrative region level variables in the multilevel model as compared to the null model, which is calculated by the difference from variance in the null model to variance in the full model divided by variance in the null model. Finally, Adjusted Odds Ratios (AOR) with a 95% Confidence Interval (CI) were reported in the best fit multivariable multilevel model. *P*-value less than 0.05 in the best fit multivariable model were used to declare significance.

## Results

### Background characteristics of the study respondents

The majority (69.7%) of study participants were living in a rural setting; more than one-third (36.34% of) under-5 children were delivered at home. Nearly, three-fourths (70.2%) of mothers aged were range between 20 and 34 years. Around 29,406 (15.3%) mothers give birth aged less than 19 years. More than 89% of mothers had at least one antenatal care follow-up during their pregnancy period (Table 1).

**Table 1:** Background characteristics of the study respondents in SSA

Variables	Categories	Frequency (n)		Un weighted percentage
		**Un weighted**	**Weighted**	
Maternal age	15–19	11,669	11,361	6.1
	20–34	134,928	134,516	70.2
	35–49	45,535	44,549	23.7
Teenage pregnancy	≥ 20 years	162,726	161,730	84.7
	≤ 19 years	29,406	28,606	15.3
Marital status	Not currently married	10,807	10,213	5.6
	Married	181,325	180,12	94.4
Mother educational status	No education	77,858	74,981	40.5
	Primary	65,972	65,433	34.4
	Secondary and above	48,302	49,923	25.1
Mother occupational status	Not-working	58,129	56,599	32.7
	Working	119,514	119,964	67.3
Husband educational status	No education	59,746	57,995	37.2
	Primary	47,407	48,145	29.6
	Secondary and above	53,299	54,022	33.2
Husband occupational status	Not-working	6,893	6,786	4.3
	Working	154,394	154,673	95.7
Residence	Urban	58,043	60,087	30.2
	Rural	134,089	130,249	69.8
Wealth status	Poor	91,653	84,402	47.7
	Middle	37,395	38,292	19.5
	Rich	63,084	67,642	32.8
Country income	Low income	124,779	125,142	64.9
	Lower middle income	61,009	59,306	31.8
	Higher middle income	6,344	5,888	3.3
Sub-Saharan region	East Africa	81,232	81,373	42.3
	West Africa	70,832	70,231	36.9
	Central Africa	37,699	36,402	19.6
	Southern Africa	2,369	2,330	1.2
**Total**		**192,132**	**190,336**	**100**

### Under-5 children characteristics

A total of 192,132 under-5 children participated in this study. Of all under-5 children, 96,892 (50.4%) of them were males. A total of 9,397 (6.2%) under-5 children were born shorter than 18 months preceding birth. Overall, 28,791(16.1%) under-5 children were low birth weight. Nearly three-fourths (61%) of under-5 children were not exclusively breastfed (Table 2).

**Table 2:** Under-5 children characteristics of SSA countries using the recent DHS 2010 to 2020

Variables	Categories	Frequency (n)		Un weighted Percentage
		**Un weighted**	**Weighted**	
Infant sex	Male	96,892	96,101	50.4
	Female	95,240	94,235	49.6
Plurality	Single	186,103	184,380	96.9
	Multiple	6,029	5,957	3.1
Preceding birth interval	≥ 24 months	123,975	122,798	81.6
	18–23 months	18,619	18,102	12.3
	< 18 months	9,397	9,119	6.2
Birth size at birth	Smaller than average	28,791	28,011	16.1
	Average	87,911	87,354	49.1
	Larger than average	62,478	62,868	34.8
Exclusive breast feeding	No	107,123	106,313	60.9
	Yes	68,767	68,881	39.1
ANC visits	No ANC	14,655	13,849	10.9
	At least one ANC	120,354	120,464	89.1
Place of delivery	Health facilities	122,131	123,246	63.6
	Home delivery	69,852	66,951	36.4
**Total**		**192,132**	**190,336**	**100**

### The pooled estimate of overweight /obesity among under-5 in SSA

**Fig. 1:**
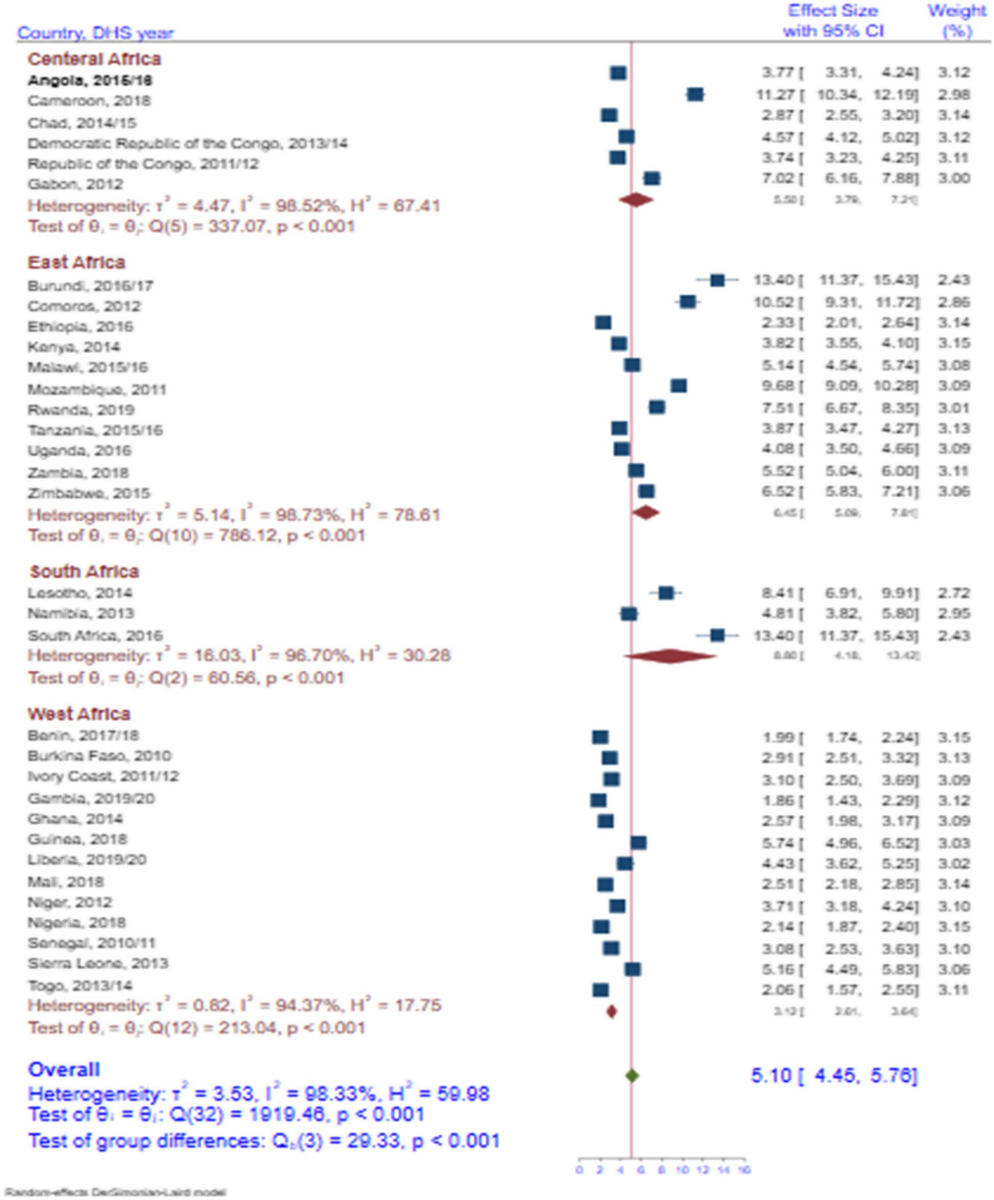
Forest plot of overweight /obesity among under-5 in SSA 2010 to 2020

The pooled prevalence of overweight /obesity among under-5 was 5.10% (9% CI: 4.45 – 5.76) in SSA with statistically significant heterogeneity. Across regions of SSA, the South Africa region (8.80%, 95% CI: 4.18 – 13.42) had a higher prevalence of under-5 children overweight and/or obesity followed by the East Africa region. The West Africa region had the lowest prevalence of under-5 children overweight and/or obesity (Fig.1).

### Determinants of overweight /obesity among under-5 children

From the multilevel mixed-effects logistic regression model, the full (both individual and region/province level factors) were the best fit model (LLR = -19,360.77). From the null model, the intraclass correlation coefficient was 12%, which indicates controlling region-level dependency is mandatory.

From the individual and region level, multivariable mixed-effects logistic regression analysis, sex of the child, birth weight, child age, educational status of the mother, place of delivery, and sub-region of SSA were the independent determinants of overweight /obesity among under-5 in SSA. The prevalence of overweight and/or obesity among under-5 children was 5% in SSA, thus it is fair to interpret the odds effect size as relative risk. Male under-5 children were 9% higher risk of developing obesity than female under-5 children (AOR = 1.09, 95 CI: 1.02 – 1.25). The risk of developing overweight and/or obesity among average birth weight under-5 children were 16% more likely as compared to low birthweight under-5 children (AOR = 1.16, 95 CI: 1.05 – 1.28). Larger than average birth weight under-5 children had a 39% higher risk of overweight and/or obesity as compared to their counterparts (AOR = 1.39, 95% CI: 1.26 – 1.54). Under-5 children aged two to three years had less risk of developing overweight and/or obesity by 15% than under-5 children younger than two years (AOR = 0.85, 95% CI: 0.76 – 0.94). Under-5 children born from educated mothers (secondary and above) were 12% higher risk of developing overweight and/or obesity as compared to under-5 children born from non-educated mothers (AOR = 1.12, 95% CI: 1.01 – 1.25). Furthermore, under-5 children born in the West Africa region were at a 33% lower risk of obesity as compared to under-5 children living in East Africa (AOR = 0.67, 95% CI: 0.56 – 0.81). While under-5 children living in South Africa 87% higher risk of developing overweight and/or obesity as compared to under-5 children born from the East Africa region (AOR = 1.87, 95% CI: 1.09 – 3.21) (Table 3).

**Table 3:** Multivariable multilevel mixed-effects logistic regression analysis factors affecting under-5 overweight and/or obesity in SSA

	**Null Model **^**a**^	**Model I **^**b**^	**Model II **^**c**^	**Model III **^**d**^
		AOR (95% CI)	AOR (95% CI)	AOR (95% CI)
**Sex of the child**				
Female		1		1
Male		1.09 (1.02 – 1.15)		1.09 (1.02 – 1.25) **
**Plurality of birth**				
Single		1		1
Multiple		0.84 (0.71 – 0.99)		0.84 (0.70 – 1.00)
**Birth size**				
Low birth weight		1		1
Average birth weight		1.16 (1.06 – 1.28)		1.16 (1.05 – 1.28) **
Larger than average		1.39 (1.26 – 1.53)		1.39 (1.26 – 1.54) ***
**Child age in years**				
Less than two years		1		1
Two–three years		0.84 (0.76 – 0.94)		0.85 (0.76 – 0.94) ***
Above three years		0.70 (0.63 – 0.78)		0.70 (0.63 – 0.78) ***
**Exclusive breastfeeding**				
Yes		1		1
No		1.00 (0.91 – 1.10)		1.00 (0.91 – 1.11)
**Preceding birth interval**				
≥ 24 months		1		1
18 -23 months		0.94 (0.85 – 1.03)		0.94 (0.85 – 1.03)
< 18 months		0.99 (0.87 – 1.12)		0.98 (0.87 – 1.12)
**Age of the mother**				
15—19 years		1		1
20—34 years		0.80 (0.63 – 1.51)		0.79 (0.63 – 1.01)
≥ 35 years		0.81 (0.64 – 1.03)		0.81 (0.63 – 1.03)
**Mother education**				
No education		1		1
Primary education		1.06 (0.97 – 1.15)		1.03 (0.95 – 1.08)
Secondary and above		1.15 (1.03 – 1.28)		1.12 (1.01 – 1.25) *
**Father education**				
No education		1		1
Primary education		1.02 (0.93 – 1.11)		0.99 (0.90 – 1.08)
Secondary and above		1.09 (0.99 – 1.21)		1.07 (0.97 – 1.18)
**Mother’s occupation**				
Not working		1		1
Working		0.95 (0.88 – 1.01)		0.94 (0.88 – 1.01)
**Father occupation**				
Not working		1		1
Working		1.18 (0.99 – 1.39)		1.18 (0.99 – 1.39)
**Family size**				
< 4		1		1
≥ 4^+^		0.98 (0.92 – 1.05)		0.98 (0.91 – 1.05)
**Wealth index**				
Poor		1		1
Middle		0.98 (0.90 – 1.06)		0.99 (0.91 – 1.08)
Richer		1.02 (0.94 – 1.01)		1.06 (0.98 – 1.16)
**Place of delivery**				
Institutional		1		1
Home		0.93 (0.86 – 0.99)		0.91 (0.85 – 1.08)
**SSA Sub-region**				
East Africa			1	1
West Africa			0.70 (0.59 – 0.84)	0.67 (0.56 – 0.81) ***
Central Africa			1.16 (0.95 – 1.42)	1.09 (0.88 – 1.35)
South Africa			2.29 (1.59 – 3.29)	1.87 (1.09 – 3.21) *
**Country income**				
Upper middle income			1	1
Lower middle income			0.97 (0.71 – 1.39)	1.04 (0.70 – 1.55)
Low income			0.76 (0.56 – 1.04)	0.82 (0.56 – 1.21)
**Residence**				
Urban			1	1
Rural			0.92 (0.87 – 0.97)	1.08 (0.99 1.18)
**Random effects**				
**ICC%**	**0.12**			
**MOR (95%CI)**	**1.92**	**1.84**	**1.79**	**1.78**
**PCV%**		**0.12**	**0.21**	**0.22**
**Model compression**				
**Log likelihood ratio**	**-32,494.5**	**-19,383.98**	**-32,453.14**	**-19,360.77**

NB: * = Significant at *P*-value 0.05, ** = Significant at *P*-value 0.01, *** = Significant at *P*-value 0.001, *CI* Confidence interval, *AOR* Adjusted odds ratio, *ICC* Intraclass correlation, *MOR* Median odds ratio, *PCV* Percentage change variation^a^, Null model^b^, Individual level variable model^c^, Community level variable model^d^, Full model

## Discussion

Overweight and/or obesity is/are important indicators of under-5 children's health. We identified independent determinant factors for under-5 children overweight and/or obesity in SSA Countries; Multivariable multilevel mixed-effects logistic regression model showed that male sex, large birth weight, macrocosmic babies, old age children, maternal education, West Africa, and South Africa region were significantly associated with under-5 children overweight and/or obesity. The pooled prevalence of under-5 overweight and/or obesity was 5.10% (95% CI: 4.45 – 5.76). This is in line with a report by the World Health Organization in 2020 that the global average prevalence of overweight/obese among under-5 children was 5.7%. However, this is lower than a study conducted in northern America (9.1%), Latin America and the Caribbean (7.5%), northern Africa (13.0%), Cameron (8%) [23], Southeast Asia (7.5%), Australia and New Zealand (16.9%) and Europe ( 8.3%) [24]. The difference might be due to socio-economic and socio-cultural differences related to nutrition across each region. As well, the reason for this discrepancy might be different cultural feeding practices and food preferences among under-5 children and different methodological approaches.

This study further tried to investigate the determinants of under-5 overweight and/or obesity. Being male had a higher risk of being under-5 overweight and/or obese. Male under-5 children were 9% higher risk of developing overweight and/or obesity than female under-5 children. This is consistent with different studies done in China [25], Brazil [26], Cameroon [23], and Ethiopia [27]. This might be driven by gender-related influences, such as social influence on body weight and parental feeding practices, as well as sex-related influences, such as body composition and hormones [28]. Regarding sex-related hormones, studies reported that females also exhibit higher circulating concentrations of leptin, a hormone that subdues appetite and promotes energy utilization, and high levels of serum leptin act on fat mass and increase levels of adiposity [29, 30] Additionally higher concentrations of androgens in males have a suppressive effect which is responsible for lower leptin serum concentrations compared with females [30, 31]. However, this result is in contrast to a study done in 12 countries (Australia, Brazil, Canada, China, Colombia, Finland, India, Kenya, Portugal, South Africa, the United Kingdom, and the United States) [32] and in a study done in Ghana shows that there is no significant gender difference to overweight and/or obesity [33].

Large birth weight had associated with an increased risk of subsequent overweight and/or obesity on under-5 children. This is similar to a study done in Brazil [26] and England [34], a systematic review and a meta-analysis from 26 Countries [35]. This association might be justified based on a research review carried out by Araújo de França et al., 2014 [36], birth bodyweight associated with overweight later in life, including greater waist and hip circumference. Babies are born macrocosmic likely due to excessive nutrition in utero, which are associated with alterations in the developing fetus that will predispose them to obesity postnatal period [37]. Most of the time increased adiposity and greater macrocosmic at birth are linked to maternal obesity and gestational diabetes mellitus [38, 39]. Fetal secretion of insulin in response to increased maternal glucose levels drives fetal growth, and increased maternal nutrient supply to the fetus will result in higher insulin levels and greater adiposity, and subsequently to developmental programming of fetal adipose tissue [40]. However, this finding was in contrast to a study reported by Andriani [41] in which children born with low birth weight increase the risk of obesity in later life.

Older age had less odds of becoming overweight and/or obese than younger age children. In this study, under-5 children aged two to three years had less risk of developing overweight and/or obesity by 15% than under-5 children younger than two years. This is similar to different studies done in Ethiopia [27], Cameroon [23], in low- and middle-income countries (LMICs) [42], and Brazil [26]. This might be due to increased physical inactivity of children less than two years that may not expose to open playgrounds associated with an increase in the chance of being overweight and/or obese.

Under-5 overweight and/or obesity had related to maternal education. Under-5 children born from educated mothers (secondary and above) were 12% higher risk of developing overweight and/or obesity as compared to under-5 children born from non-educated mothers. This is consistent with a study done in South Asia [43]. This might be probably because less educated mothers may have low levels of nutrition literacy. They might rightward shift in consumption of junky foods (foods that are rich in saturated fats, refined carbohydrates, and sweetened carbonated beverages) with low levels of polyunsaturated fatty acids and fibers. These predispose their babies to obesity and/or overweight. This implies that maternal education is important to play a significant role in reducing under-5 overweight and/or obesity.

Furthermore, under-5 children living in West Africa were at 33% lower risk of obesity as compared to under-5 children born in the East African region. Besides, under-5 children born in the South Africa region 87% higher risk of developing overweight and/or obesity as compared to under-5 children born in the East Africa region. This is similar to the World Health Organization report that there is a high prevalence of overweight and/or obese children under-5 years in southern Africa (12.1%) than in Eastern Africa (4.0%) [24].

This study was conducted based on a nationally representative multi-country dataset that could enhance the generalizability across the region. Controlling data dependency using multilevel analysis would give unbiased effect size estimates. However, the data were collected cross-sectionally using self-reported interviews, which would be prone to recall and social desirability bias.

## Conclusion and recommendations

Childhood obesity is becoming a great challenge and double burden in developing nations. Male under-5 children, older aged under-5 children, under-5 children born from educated mothers, and under-5 children living in the South Africa region were at high risk for developing overweight and/or obesity, whereas under-5 children living in West Africa region. Thus, respective countries should give primary attention to under-5 obesity, which is becoming a double burden in the region. So, early intervention is warranted in SSA to pause the consequences of the double burden of undernutrition.

## Data Availability

The datasets used and/or analyzed during the current study are available from the corresponding author on reasonable request.
